# An instrument to measure job satisfaction of nursing home administrators

**DOI:** 10.1186/1471-2288-6-47

**Published:** 2006-10-09

**Authors:** Nicholas G Castle

**Affiliations:** 1 A649 Crabtree Hall, Graduate School of Public Health, 130 DeSoto Street, Pittsburgh, PA 15261, USA

## Abstract

**Background:**

The psychometric properties of the nursing home administrator job satisfaction questionnaire (NHA-JSQ) are presented, and the steps used to develop this instrument.

**Methods:**

The NHA-JSQ subscales were developed from pilot survey activities with 93 administrators, content analysis, and a research panel. The resulting survey was sent to 1,000 nursing home administrators. Factor analyses were used to determine the psychometric properties of the instrument.

**Results:**

Of the 1,000 surveys mailed, 721 usable surveys were returned (72 percent response rate). The factor analyses show that the items were representative of six underlying factors (i.e., coworkers, work demands, work content, work load, work skills, and rewards).

**Conclusion:**

The NHA-JSQ represents a short, psychometrically sound job satisfaction instrument for use in nursing homes.

## Background

Job satisfaction is defined as "the favorableness or unfavorableness with which employees view their work" [1]. Some recent research would suggest that job satisfaction of employees within an organization is related to an organization's ability to change [2]. Since a consistent theme in the literature for the past 20 years (or more) has been the inability of some nursing homes to change in a meaningful way, especially in the area of quality of care [3], in this context improving job satisfaction may be important in improving some aspects of the industry.

Job satisfaction of nursing home administrators (NHAs) may be especially important, because administrators can have a pervasive influence on facility performance and quality of care [4]. Castle [5], for example, has shown a positive association between NHA turnover and the resident outcomes of catheterization, restraint use, pressure ulcers, psychoactive medications, and quality of care deficiencies. Smith, Shortell, and Saxberg describe NHAs as "the critical variable affecting quality of care" [6]. Singh and Schwab [7] have examined the organizational destabilization that can occur when NHAs turnover [7]. Resident satisfaction would also appear to be influenced by NHAs [8]. Given the importance of NHAs and their potential impact on quality, a valid and reliable instrument to assess their job satisfaction is desirable. However, we found that no instrument currently exists that was developed specifically for this purpose. In this investigation, we use data from 721 NHAs to develop such an instrument.

Several generic job satisfaction instruments are readily available for use [9]. These include the Job Description Index [10], revised Index of Work Satisfaction [11], modified Job Description Index [12], and the Measure of Job Satisfaction [13], to name just a few. Gillies, Foreman, and Pettengill [14] reviewed job satisfaction instruments, and found none to be extensively used or developed with long-term care settings in mind. This by itself is not necessarily problematic, as these instruments were designed for general applications. But what is problematic is that studies using existing job satisfaction instruments in long-term care settings have generally been dissatisfied with the performance of these preexisting instruments [15].

Moreover, other factors may also reduce the performance of these preexisting instruments for use with NHAs. NHAs work in a fairly unique environment, with a flat administrative structure, large number of unskilled workers, and high turnover. That is, the determinants of NHAs job satisfaction may not be different from that of other professionals, but existing instruments may not be sensitive to the unique work environment. A flat administrative structure may reduce the number of professional peers with whom NHAs can collaborate. NHAs may be professionally isolated (i.e., coworkers may be an important influence on NHAs' job satisfaction). Moreover, empirical analyses [16] have shown the flat administrative structure to increase the work load of NHAs. In many instances, to accomplish objectives, NHAs must undertake initiatives themselves (i.e., work demands may be an important influence on NHAs' job satisfaction). The large number of unskilled workers may not adequately match the NHAs' work skills. For example, NHA licensure includes knowledge of quality assurance and regulations; however, the unskilled workers that constitute a vast majority of the workforce may be more concerned with staffing schedules and the social climate (i.e., work skills may be an important influence on NHAs' job satisfaction). Both unskilled workers and other top managers (i.e., the Director of Nursing) characteristically have high levels of turnover. This high staff turnover may reduce teamwork (i.e., teamwork may be an important influence on NHAs' job satisfaction). This unique environment would seem to indicate that a specific NHA job satisfaction instrument could be useful. Also, anecdotal evidence from hundreds of contacts with NHAs in the recent past, suggested to us that a specific NHA job-satisfaction instrument could better capture job satisfaction than preexisting instruments.

Studies identified examining job satisfaction of caregivers in long-term care settings are shown in Table 1. The results (Table 1) show job satisfaction is clearly important for caregivers in nursing homes. For example, in six of the 19 studies listed, lower job satisfaction was associated with turnover/absenteeism. However, we do note that the vast majority of these studies have not examined NHAs. These previous studies have used differing job satisfaction instruments. Also, it is clear that existing job satisfaction instruments use relatively few satisfaction subscales. Moreover, no instrument included all of the job satisfaction items/subscales identified above as potentially important for NHAs (i.e., coworkers, work demands, work skills, and teamwork).

**Table 1:** Summary of Job Satisfaction Studies in Long-Term Care Settings

**Author(s)**	**Job Satisfaction Instrument**	**Job Satisfaction Domains**	**Sample Size and Setting**	**Significant Findings**
Murphy (2004) [30]	Job Descriptive Index (JDI)	Work on present job Pay	149 nursing home administrators in Iowa	Most dissatisfied with coworkers and pay
		Opportunities for promotion		
		Supervision		
		Co-workers		
		Job in general		
Parsons et al. (2003) [31]	Modified from Herzberg (1966)	Personal opportunity Supervision	550 NAs in 70 facilities in Louisiana	Most dissatisfied with pay, benefits, and recognition
		Benefits		
		Coworker support		
		Social rewards		
		Task rewards		
Moyle et al. (2003) [32]	N/A	Workplace flexibility	27 RNs and NAs in one facility in Australia	Satisfaction was linked to workplace flexibility, residents, team environment, and better resident care
		Team environment		
		Optimal resident care		
Chou, Boldy, & Lee (2002) [13,33]	Measure of Job Satisfaction (MJS)	Professional support	Seventy facilities with 610 nursing home staff and 373 hostel care staff in Australia	Job satisfaction is associated with professional support
		Personal satisfaction		
		Workload		
		Training		
		Team spirit/co-workers		
Will & Simmons (1999) [34]	Job Descriptive Index (JDI)	Work on present job Pay	423 NAs in 29 nursing homes in Ohio	Satisfied most with work and least with pay
		Opportunities for promotion		
		Supervision		
		Co-workers		
		Job in general		
Atchison (1998) [35]	Job Diagnostic Survey	Satisfaction	283 NAs in 24 nursing homes	Job satisfaction lowest for security, growth/development, socialization, and challenges
		Job security		
		Coworkers		
		Sense of accomplishment		
		Helping other people		
		Dissatisfaction		
		Pay/benefits		
		Potential for job growth		
		Management		
		Autonomy		
Kiyak, Namazi, & Kahana (1997) [36]	Job Descriptive Index (JDI)	Work on present job Pay	308 nursing home and community agency staff	Higher dissatisfaction associated with turnover
		Opportunities for promotion		
		Supervision		
		Co-workers		
		Job in general		
Gillies, Foreman, & Pettengill (1996) [14]	Index of Work Satisfaction (IWS)	Autonomy Interaction	44 nurse directors and nurse educators working in long-term care facilities	Job satisfaction highest for interactions, autonomy, and professional status
		Agency policies Pay		
		Professional status		
		Task requirement		
Grieshaber, Parker, & Deering (1995) [1]		Work environment	Two nursing homes	
		Job content		
Irvine & Evans (1995)+ [6]	N/A	Routinization	Meta-analyses with combined sample size of 5,352	Work content and work environment are more strongly associated with job satisfaction than economic variables
		Autonomy		
		Feedback		
		Role conflict		
		Role ambiguity		
		Work overload		
Coward et al. (1995) [15]	Modified Stamps and Piedmonte (1986) scale [IWS]	Professional status	281 RNs and LPNs from 26 nursing homes	Five factors associated with job satisfaction (race, income, supervisor, initial intent to stay, current intent to leave)
		Task requirement		
		Autonomy		
		Interactions with other nurses		
		Pay		
Monahan & Carthy (1992) [37]	N/A	Attachment	75 NAs at 7 nursing homes	Attachment most related to retention of NAs
		Gratification		
		Demands		
		Monetary needs		
		Decision-making		
Grau et al. (1991) [38]	Combined several scales	Job process	219 NAs in one nursing home	Social atmosphere and job benefits associated with institutional loyalty
		Attitudes toward administration		
		Social atmosphere		
		Job benefits		
		Job tasks		
Anderson, Aird, & Haslam (1991) [39]	NG	None	212 nursing staff in 6 nursing homes	Nursing staff have high levels of satisfaction, but is associated with absenteeism
Humphris & Turner (1989) [40]	Porter (1962) scale	Working conditions	84 nurses at a unit for the elderly severely mentally infirm	Low satisfaction was associated with turnover from unit
		Emotional climate		
		General		
Mullins et al. (1988) [41]	Job Satisfaction Survey (JSS)	Pay	Heads of departments (n = 439) from 46 nursing homes	Most satisfied when individual efforts are rewarded
		Promotion		
		Supervision		
		Benefits		
		Rewards/appreciation		
		Working conditions		
		Coworkers		
		Nature of job		
		Communication		
Deckard, Hicks & Rountree (1986) [42]	Job Diagnostic Survey (JDS)	Skill variety	340 nurses from a nursing home chain	Job satisfaction was similar to norms in other occupations
		Task identity		
		Task significance		
		Autonomy		
		Job feedback		
Waxman et al. (1984) [43]	Minnesota Satisfaction Scale	Job Satisfaction Scale	234 NAs in 7 facilities, uses 20 questions for overall job satisfaction score	Positive association between job satisfaction and turnover
Bergman et al. (1984) [44]	None	Job	12 long-term care facilities and 432 RNs, LPNs, and NAs	Descriptive results provided
		Knowledge, skill, and attitudes		
		Autonomy		
		Stress		

NA = Nurse Aide; RN = Registered Nurse; LPN = Licensed Practical Nurse.

+ = This study is a meta-analysis, and does not include only long-term care studies.

NG = Not given; N/A = Not applicable.

In this case, the use of relatively few satisfaction subscales in existing instruments was important for a second reason. That is, since we know little regarding NHAs' job satisfaction, we sought to identify a wide range of subscales; thus providing us with more information. We also found some job satisfaction instruments used dichotomous response options. We were interested in using an instrument with multiple response categories, so that we could examine the relative degree of satisfaction and dissatisfaction of NHAs, which of course is not possible using a dichotomous scale. Many existing job satisfaction instruments use a relatively large number of questions. To increase the likelihood that the final instrument would be used by the industry, we considered it important to minimize the number of questions. It was because of these more-desirable instrument requirements, and the general unsuitability of existing instruments, that we developed the nursing home administrator job satisfaction questionnaire (NHA-JSQ).

## Methods

### Job satisfaction questionnaire

#### Subscales

In developing the NHA-JSQ, our first consideration was to determine what areas of concern the questions should address (i.e., subscales). Therefore, using open-ended questions and mail surveys, we asked 93 administrators to list specific areas of their jobs that they believed were most (least) important, and gave them most (least) satisfaction. That is, we believe that NHAs receive satisfaction from their job activities. This follows the "met expectations" theory of job satisfaction, whereby job satisfaction is believed to be the result of the agency between the individual and employer [17]. We used this approach, because we wanted an instrument that was both theoretically grounded and psychometrically sound.

The responses were transcribed and then collapsed into similar areas/themes by using content analysis [18]. We excluded variables that seemed more likely to moderate job satisfaction rather than be a dimension of job satisfaction. For example, concerns regarding federal and state regulations were not included, although many NHAs voiced concerns in these areas. A list of 11 candidate subscales was identified, and an additional 73 NHAs in a mail survey were asked to rate the top five areas. After taking the average score for each subscale, we found a drop in the over-all rating for the seventh subscale. Based on this result, we considered six areas of job satisfaction to be most important from the perspective of NHAs.

Clearly, what is included in each domain depends somewhat on the definition used to operationalize each domain. We attempted to label our six domains (and defined each domain) to be consistent with prior studies [19]. This process included examining the domains (and their definitions) of the studies listed in Table 1. We labeled the domains as: Coworkers, Work Demands, Work Content, Work Load, Work Skills, and Rewards.

'Coworkers' represents relations with other workers in the facility; 'Work Demands' represents resources and demands of the job; 'Work Content' represents the complexity and challenges of the work; 'Work Load' represents time pressures; 'Work Skills' represents preparation for the position; and, 'Rewards' represents benefits of the job.

#### Item development

To develop items for the NHA-JSQ, we examined questions from previously published job satisfaction instruments (n = 237 questions) and specific comments provided by NHAs in the mailings described above (n = 83 comments). Items that seemed to fit the six subscales listed above were chosen, and these items were rewritten: (1) to conform to the scaling requirements of the survey (described below); (2) to be relevant to NHAs (i.e., face validity); and (3) to be relevant to the nursing home context (i.e., content validity). We then had a six-member research team, consisting of experts and practitioners in survey development, gerontology, geriatrics, and long-term care, choose their candidate items from this initial item pool. These experts were asked to pick three questions in each satisfaction subscale, which they thought best captured the information. The five most highly rated questions in each area were included in our pilot instrument.

#### Response scale

Examining five different types of response scales, recent research has shown that a visual analogue rating scale from 1 to 10 (i.e., a graphic scale) was both the most-popular scale among elders, and was least prone to response bias [20]. This prior study was conducted on community-dwelling elders, and not NHAs. Nevertheless, the issue of lack of response variability is common to both job satisfaction [6,17] and elder satisfaction surveys. Therefore, we also investigated the use of this graphic scale with NHAs. In face-to-face interviews we conducted with NHAs (N = 27), they preferred a 1 to 10 graphic rating scale; therefore, this response scale was used in the NHA-JSQ.

### Sources of data

Data used in this investigation to validate the pilot NHA-JSQ described above came from a mail survey of NHAs. The NHA-JSQ was mailed to 1,000 NHAs located in two states, New York (NY) and Pennsylvania (PA). These states were chosen as a convenient sample, and only two states were used because we had limited resources for this research and we needed to limit the sample size.

For this survey mailing, a random sample of approximately 70 percent of facilities was chosen from each state's pool of eligible facilities. Eligible nursing homes were defined as those participating in Medicare and/or Medicaid certification, which includes approximately 97 percent of all nursing homes. We used this eligibility definition because these are the nursing homes included in the On-line Survey, Certification, and Reporting (OSCAR) system data, which was used to identify the mailing address of these facilities. At the time of this study (spring 2004), eligible facilities included 673 nursing homes from NY and 749 from PA. In addition, we excluded owners from the analyses (N = 32), but this could only be done after data collection, using a self-reported measure. These owner/administrators were excluded because they are likely able to influence their work experience, including schedule, pay, and work content.

The mailing to NHAs included the survey, letter of introduction, and postage-paid reply envelope. In addition, we included a manuscript we had published in a nursing home trade magazine. Included in the letter of introduction, we indicated that a similar manuscript would be written from the current survey, and aggregate results could be sent directly to the administrator.

The OSCAR was also used as a minor source of data, primarily for descriptive analyses. The OSCAR is conducted by state licensure and certification agencies as part of the Medicare/Medicaid certification process, and includes almost all nursing homes in the U.S. (see [21] for a more extensive description of this data). Despite some potential validity and reliability issues (see [22]), the OSCAR data is widely used. For example, the OSCAR is often used by researchers as a secondary source of nursing home characteristics [3]. Given that the OSCAR was a minor source of data for our analyses, these validity/reliability issues with the OSCAR data were of little consequence for the analyses presented.

### Analyses

Descriptive analyses are first presented consisting of the percent or mean for characteristics of the NHAs and characteristics of the nursing homes in the sample. In addition, we used bivariate comparisons for respondent and non-respondent facilities using the OSCAR data.

The NHA characteristics are self-evident, and are not described further. The nursing home characteristics used are bed size, ownership, chain membership, Medicaid occupancy, overall-resident census, case mix (using Activities of Daily Living [ADLs] and dementia), deficiency citations, staff turnover, and staffing levels. The number of beds in the facility is used as a measure of facility size. Two classes of facility ownership are used, for-profit and not-for-profit. Public facilities, such as state and locally run nursing homes, represent a minor market presence and were thus included in the not-for-profit class of ownership. Two classes of chain membership are also used, chain and non-chain. Medicaid occupancy represents the number of residents paid for by the Medicaid program, divided by the total number of all residents (multiplied by 100 to create a percent). Average resident census represents the total number of residents divided by the total number of beds (multiplied by 100 to create a percent). For each of three ADL questions (eating, toileting, and transferring) in the OSCAR, we assign a score from 0 to 3 by using no assistance, moderate need for assistance, and high degree of need for assistance, respectively. We then sum these scores. Higher scores indicate a greater average ADL impairment within the facility. Dementia represents the number of residents with dementia, divided by the total number of all residents (multiplied by 100 to create a percent). Deficiency citations are a count of the number of citations given to the facility in the most-recent licensure/certification survey. Staff turnover was determined using the percent of staff leaving the facility (voluntary or involuntary) during the previous year. Staffing levels represent the number of full-time equivalent (FTE) staff employed per 100 beds, and includes full-time, part-time, and temporary staff.

The characteristics of the NHAs and characteristics of the nursing homes in the sample were used to provide descriptive statistics. The NHAs' responses to the job satisfaction items were used to develop the NHA-JSQ. In this development process, the first analytic objective was to reduce the larger set of items to a smaller set, such that this smaller set of items would adequately represent the factor structure. The second objective was to report the applied psychometric properties of the NHA-JSQ.

Factor analyses were used to test the extent to which the items in each domain appeared to represent the same underlying construct; that is, to measure the degree of congruence between the domains of interest and the questions used to measure these attributes. Varimax rotation was used and a factor-loading criterion of .40 and uniqueness of < .90 were used to retain items.

As McHorney, Ware, Lu, and Sherbourne [23] point out, in many cases reporting standard psychometric properties may still not be sufficient in many cases to make an accurate judgment regarding the actual performance of an instrument when in use. These authors recommend reporting the applied psychometric properties of instruments, such as the completeness of data, score distributions (i.e., ceiling and floor effects), item-scale consistency, and reliability of domain scores. Following these recommendations, the percent of NHAs' not providing responses for each question was determined. This information on non-response is important because a score for each scale cannot be confidently computed if a high number of individual items comprising that scale is missing [23]. Score distributions include floor and ceiling effects; although, for job satisfaction scores, ceiling effects are usually of most interest. In our case, these are calculated by reporting the percent of responses with a rating of 1 (floor) and 10 (ceiling). Item-scale internal consistency was determined, using Cronbach's alpha for each subscale, and represents the degree to which items correlate within each subscale.

## Results

Seven hundred and twenty-one responses were received from non-owner NHAs (response rate = 72 percent). The response rate varied little across the states, with PA having a response rate of 74 percent (n = 337) and NY 69 percent (n = 295). Most (76 percent) of the questionnaires were returned by mail within one month. Also, because we were able to link facilities with OSCAR data, we determined that no significant differences on facility characteristics (i.e., bed size, ownership, chain membership, Medicaid census, and staffing levels) existed for respondents, compared to non-respondents.

Table 2 presents descriptive statistics of NHAs, along with facility characteristics. Similar to other studies in this area, NHAs were most likely to have a Masters degree or higher (67 percent), and be a member of a professional society (91 percent). No differences between the state samples were observed (not reported).

**Table 2:** Descriptive Statistics of Nursing Home Administrators and Nursing Homes

**Characteristic**	**Percent or Mean (Std.)**
*Nursing Home Administrators*	
Gender (Female)	54%
Age (years)	52 (8.6)
Highest level of education:	
High School	25%
Bachelors degree	8%
Masters degree or higher	67%
Member of professional society/organization	91%
Number of places employed as an administrator	7.4
Tenure as administrator in current facility (years)	4.7 (4.3)
Tenure as an administrator (years)	16.9 (9.6)
	
*Nursing Homes*	
Organizational size	137 (102)
For-profit ownership	46%
Chain membership	35%
Medicaid occupancy	57%
Average census	91%
Resident ADLs	1.25 (0.98)
Dementia	0.47 (0.18)
Deficiency citations	4.36 (3.72)
RN turnover	76%
LPN turnover	78%
NA turnover	107%
FTE RNs per 100 beds	9.76 (7.61)
FTE LPNs per 100 beds	14.06 (9.54)
FTE NAs per 100 beds	36.36 (9.06)

FTE = full-time equivalent; RN = Registered Nurse; LPN = Licensed Practical Nurse; NA = Nurse Aide; ADL = Activities of Daily Living.

Table 3 presents descriptive statistics and psychometric properties of the NHA-JSQ. This consists of 18 items. Cronbach's alphas for the domains are shown in the first column, and all were higher than the usually recommended level of 0.70 [23]. The primary factor loadings from the factor analyses are shown in the second column. All loadings exceeded the minimum cutoff of 0.40, indicating that the items were representative of the underlying factors. In addition, the groupings of items (i.e., emergent factor structure) were the same as those proposed in the pilot instrument, as shown by the eigenvalues greater than 1.0, indicating a single factor solution for each domain. These eigenvalues were 4.32 (coworkers), 3.37 (work demand), 2.26 (work content), 2.02 (workload), 1.82 (work skills), and 1.69 (rewards).

**Table 3:** Psychometric Properties of the Nursing Home Administrator Job Satisfaction Questionnaire (N = 721)

**Item/Domain^1,2^**	**Cronbach's alpha coefficients**	**Primary Factor Loadings**	**Missing responses**	**Floor**	**Ceiling**	**Mean**	**Standard deviation**	**Item-Scale correlation**
**Coworkers**	0.74							
Rate the people you work with		0.75	0.28%	4.9%	4.3%	6.54	1.67	0.69
Rate whether you feel part of a team effort		0.73	0.14%	0.4%	1.4%	7.23	1.82	0.62
Rate co-operation among staff		0.70	0.28%	3.3%	1.1%	7.69	2.14	0.58
Rate whether staff place reasonable demands on you		0.70	0.14%	0.14%	7.6%	3.87	2.46	0.58
**Work Demands**	0.78							
Rate the support available to you in your job		0.60	0.27%	10.6%	18.0%	7.28	1.52	0.83
Rate the opportunities you have to discuss your concerns		0.60	0.27%	1.4%	4.3%	6.72	1.46	0.81
Rate the demands residents and family place on you		0.60	0.14%	4.2%	9.7%	4.98	2.12	0.79
Rate whether you feel you are doing a good job		0.57	0.28%	0.56%	7.2%	7.44	1.54	0.72
**Work Content**	0.72							
Rate how much you enjoy working with residents		0.61	0.14%	0.28%	1.7%	6.81	1.97	0.71
Rate how your role influences the lives of residents		0.60	0.14%	5.3%	1.4%	7.26	2.29	0.69
Rate your closeness to residents and families		0.56	0.28%	5.1%	12.9%	8.04	1.64	0.64
Rate the amount of autonomy you have		0.51	0.28%	0.14%	3.7%	6.75	1.99	0.56
**Work Load**	0.73							
Rate your workload		0.58	0.27%	0.14%	11.5%	7.09	2.58	0.72
Rate your work schedule		0.54	0.14%	0.27%	6.3%	6.98	2.59	0.72
**Work Skills**	0.70							
Rate whether the demands of your job are compatible with your work skills		0.52	0.14%	0.42%	14.0%	7.69	2.14	0.60
Rate the adequacy of the training you have to perform your job		0.51	0.27%	0.27%	19.3%	8.54	1.31	0.60
**Rewards**	0.80							
Rate how fairly you are paid		0.72	0.14%	0.42%	14.0%	7.69	2.14	0.60
Rate your chances for further advancement		0.71	0.27%	0.27%	19.3%	8.54	1.31	0.60

1. All questions used a 10 point visual analogue rating format scale:

2. To avoid response set bias, the questions in the Nursing Home Administrator Job Satisfaction Questionnaire were presented in a random order, and did not include domain headings.

The percent of NHAs not providing responses for each question was low, and averaged less than 1.0 percent. The means and standard deviations show that in several cases the distributions are slightly skewed to the positive end of the scales. Although it should be noted that, for all questions, the full range of scores was used (results not shown). The floor effects are negligible; whereas, the ceiling effects were slightly higher. The lowest ceiling effect score was 1.1 percent and the highest 19.3 percent.

The item-scale internal consistency analyses (corrected for overlap) show that the correlation of items within indexes were in all cases higher than those with other indexes (not shown). McHorney et al. [23] recommend use of a measure of correlation of greater than .40 in item-scale internal consistency analysis, and our items achieved or exceed this level in all cases.

## Discussion

Due to the importance of nurses in clinical outcomes, The American Nurses Association (ANA) has advocated the development and use of a nurse-sensitive job satisfaction measure [24]. That is, a job satisfaction measure that is more relevant to nurses. The ANA believes that a job satisfaction instrument more sensitive to the nurses' work environment may be of greater use in work-improvement activities. The same may also be true for NHAs. As described previously, job satisfaction of NHAs may have important implications for quality of care and their job environment would seem somewhat unique. Due to the importance of NHAs in the nursing home, we advocate the use of an administrator-sensitive job satisfaction measure.

The NHA-JSQ represents a first step in the development of an administrator-sensitive job satisfaction instrument. The NHA-JSQ uses a simple graphic scale and is purposefully brief. This response scale and limited number of questions enables easy use by providers. Our experience shows that a typical respondent will take less than ten minutes to answer the survey questions. For users, data entry takes less than three minutes for each returned survey. Although, even with the NHA-JSQ, some users may still find it useful to receive instructions on using this instrument.

This point brings up the issue of who would use the NHA-JSQ. Individual NHAs may receive some benefit from understanding their own job satisfaction, relative to others in the field. More utility from the instrument may come from corporate entities using this instrument, and using the results to improve working conditions. Clearly, with a new instrument, relative or normative job satisfaction information is not available; although, our descriptive results do provide a starting point in this regard, providing information from a large sample of NHAs.

Some recent publications have highlighted the importance of NHAs in nursing homes (e.g., [5,25]). These studies show NHAs influence quality of care and turnover of other staff. Moreover, in a qualitative study McCarthy and Friedman [25] show NHAs are themselves sensitive to the work environment. The NHA-JSQ, in future studies, could further expand this line of research.

The domains included in the NHA-JSQ would seem to follow Herzberg's two-factor theory [26]. That is, this prior work suggested that employee motivation and satisfaction was based on hygiene factors and motivation factors, respectively. Hygiene factors include wages, inter-personal relations, and supervision. Motivation factors include responsibility, gaining recognition, and opportunity for advancement. Almost all of these factors are included in the NHA-JSQ. Herzberg further reasoned that factors causing satisfaction were different from those causing dissatisfaction, and that favorable hygiene factors are needed to avoid dissatisfaction and favorable motivation factors to provide satisfaction [26]. Future studies could examine these factors in NHAs, and whether the two factors influence the performance of administrators.

In addition, applicant attraction theories would also suggest that factors promoting job satisfaction can be significant for attracting new employees to the organization. That is, these theories would suggest that the job satisfaction of current employees is a signal influencing prospective future employees. Thus, further examining job satisfaction of NHAs may be useful for at least two reasons; first, an influx of new administrator talent into nursing homes may be beneficial; and second, we are experiencing a decline in numbers of qualified NHAs [27].

### Limitations

The NHA-JSQ may be subject to some limitations. For example, no negatively worded items were used, so response-set bias may result (i.e., respondents using the same response for all categories). In addition, in developing this instrument, administrators may have provided socially appropriate responses. Without information from an existing instrument, it is also difficult to determine how our questionnaire performs, relative to the other generic instruments described previously. Results using classical test theory are sample-dependent. Item response theory (IRT) may provide further information on the properties of the NHA-JSQ [28].

We believe one benefit of the NHA-JSQ comes from the use of few questions and simple nature of the questions. The use of few questions and simple wording leads to the use of somewhat terse language; nevertheless, this approach was used with some success for elders (e.g., [20]), with apparently greater understanding and high response rates. Similar benefits for job satisfaction and NHAs are yet to be determined. Nevertheless, we should note that in on-going work with this instrument: response rates have been high (>70 percent); NHA interviews have determined that the terse language is welcomed and extremely well understood; and, the graphic 1 to 10 response scale is liked.

In addition, we must be careful in addressing causal order. We maintain that NHA job satisfaction influences organizational performance, ability to change, and quality of care. However, the reverse may also be the case. That is, the causal order is ambiguous in these relationships.

## Conclusion

Despite the limitations described above, we believe the NHA-JSQ provides a sound instrument for use in nursing homes. We have produced a short, psychometrically sound instrument. This is important because we were unable to find in the published literature an appropriate instrument for use in this long-term care setting.

Job satisfaction of NHAs has important implications for nursing home staff, the nursing home industry, and quality of care. Other studies have shown strong linkages between job satisfaction (dissatisfaction) and job performance and turnover [29]. The NHA-JSQ could be used to examine similar relationships for administrators (i.e., job (dis)satisfaction and job performance and turnover). Moreover, it is clear that NHAs have a pivotal position in nursing homes. It is our hope that the NHA-JSQ be used, first, to further show the importance of NHAs in improving nursing home care, and second, to further improve the working conditions for NHAs themselves.

## Competing interests

The author(s) declare that they have no competing interests.

## Authors' contributions

Nicholas Castle completed all of the work in this manuscript

## Pre-publication history

The pre-publication history for this paper can be accessed here:



## References

[B1] Grieshaber LD, Parker P, Deering J (1995). Job satisfaction of nursing assistants in long-term care. The Health Care Supervisor.

[B2] Eaton SC (2000). Beyond 'unloving care:' Linking human resource management and patient care quality in nursing homes. International Journal of Human Resources Management.

[B3] (2001). Institute of Medicine. Quality of Care in Nursing Homes.

[B4] Singh DA, Amidon RL, Shi L, Samuels ME (1996). Predictors of quality of care in nursing facilities. Journal of Long-Term Care Administration.

[B5] Castle NG (2001). Administrator turnover and quality of care in nursing homes. The Gerontologist.

[B6] Smith HL, Shortell SM, Saxberg BO (1977). Administration: The critical long-term care variable. Health Care Management Review, Fall.

[B7] Singh DA, Schwab RC (1998). Retention of administrators in nursing homes: what can management do?. The Gerontologist.

[B8] Ejaz FK, Straker JK, Fox K, Swami S (2003). Developing a satisfaction survey for families of Ohio's nursing home residents. The Gerontologist.

[B9] Irvine DM, Evans MG (1995). Job satisfaction and turnover among nurses: Integrating research findings across studies. Nursing Research.

[B10] Smith PC, Hulin CL, Kendall LM, Locke EA, Fleishman EA, Bass AR (1974). The development of a method of job satisfaction: The Cornell studies. Studies in Personnel and Industrial Psychology.

[B11] Stamps PL, Piedmonte EB (1986). Nurses and work satisfaction: An index for measurement.

[B12] Boyt TE, Lusch RF, Naylor G (2001). The role of professionalism in determining job satisfaction in professional services. Journal of Service Research.

[B13] Chou SC, Boldy DP, Lee AH (2002). Measuring job satisfaction in residential aged care. International Journal of Quality in Health Care.

[B14] Gillies DA, Foreman M, Pettengill MM (1996). Satisfaction of nurse managers in long-term care. Journal of Gerontogical Nursing.

[B15] Coward RT, Hogan TL, Duncan RP, Horne CH, Hilker MA, Felsen LM (1995). Job satisfaction of nurses employed in rural and urban long-term care facilities. Research in Nursing and Health.

[B16] Castle NG, Banaszak-Holl JC (2003). The effect of administrative resources on care in nursing homes. Journal of Applied Gerontology.

[B17] Best MF, Thurston N (2004). Measuring nurse job satisfaction. Journal of Nursing Administration.

[B18] Patton MQ (2002). Qualitative Research and Evaluation Methods.

[B19] Blegen MA (1993). Nurses' job satisfaction: A meta-analysis of related variables. Nursing Research.

[B20] Castle NG, Engberg J (2004). Response scales and satisfaction of elders. The Gerontologist.

[B21] Harrington C, Carrillo H (1999). The regulation and enforcement of federal nursing home standards, 1991–1997. Medical Care Research and Review.

[B22] Straker J (1999). Reliability of OSCAR occupancy, census, and staff data: A comparison with the Ohio Department of Health annual survey of long-term care facilities. Technical Report Series 3-01: Scripps Gerontology Center, Oxford OH.

[B23] McHorney CA, Ware JE, Lu R, Sherbourne CD (1994). The MOS 36-item short-form health survey (SF-36): III Tests of data quality, scaling assumptions, and reliability across diverse patient groups. Medical Care.

[B24] American Nurses Association (2002). American Nurses Association staffing survey, 2001. http://nursingworld.org/staffing/ana_pdf.pdf.

[B25] McCarthy J, Friedman LH (2006). The significance of autonomy in the nursing home administrator profession: A qualitative study. Health Care Management Review.

[B26] Herzberg F, Mausner B, Snyderman B (1959). Motivation to Work.

[B27] Pratt J (2002). The disappearing administrator: Results of a national survey. Nursing Home and Long Term Care Management.

[B28] Hulin CL, Drasgow F, Parsons CK (1983). Item response theory: Applications to psychological measurement.

[B29] Judge T, Thoresen C, Bono JE, Patton G (2001). The job satisfaction-job performance relationship: A qualitative and quantitative review. Psychological Bulletin.

[B30] Murphy B (2004). Nursing home administrators' level of job satisfaction. Journal of Healthcare Management.

[B31] Parsons SK, Simmons WP, Penn K, Furlough M (2003). Determinants of satisfaction and turnover among nursing assistants. The results of a statewide survey. Journal of Gerontological Nursing.

[B32] Moyle W, Skinner CJ, Rowe G, Gork C (2003). Views of job satisfaction and dissatisfaction in Australian long-term care. Journal of Clinical Nursing.

[B33] Chou SC, Boldy DP, Lee AH (2002). Staff satisfaction and its components in residential aged care. International Journal for Quality in Health Care.

[B34] Will K, Simmons J (1999). Ohio CNAs speak out. Enjoyment ranks high, pay low in satisfaction study. Provider.

[B35] Atchison JH (1998). Perceived job satisfaction of nursing assistants employed in Midwest nursing homes. Geriatric Nursing.

[B36] Kiyak HA, Namazi KH, Kahana EF (1997). Job commitment and turnover among women in facilities serving older persons. Research on Aging.

[B37] Monahan RS, McCarthy S (1992). Nursing home employment: The nurse's aide's perspective. Journal of Gerontological Nursing.

[B38] Grau L, Chandler B, Burton B, Kolditz D (1991). Institutional loyalty and job satisfaction among nurse aides in nursing homes. Journal of Aging and Health.

[B39] Anderson MA, Aird TR, Haslam WB (1991). How satisfied are nursing home staff?. Geriatric Nursing.

[B40] Humphris GM, Turner A (1989). Job satisfaction and attitudes of nursing staff on a unit for the elderly severely mentally infirm, with change of location. Journal of Advanced Nursing.

[B41] Mullins L, Nelson C, Busciglio H, Weiner H (1988). Job satisfaction among nursing home personnel: The impact of organizational structure and supervisory power. Journal of Long-Term Care Administration.

[B42] Deckard GJ, Hicks LL, Rountree BH (1986). Long-term care nursing: How satisfying is it?. Nursing Economics.

[B43] Waxman HM, Carner EA, Berkenstock G (1984). Job turnover and job satisfaction among nursing home aides. The Gerontologist.

[B44] Bergman R, Eckerling S, Golander H, Sharon R, Tomer A (1984). Staff composition, job perceptions, and work retention of nursing personnel in geriatric institutions. International Journal of Nursing Studies.

